# Correlation Analysis of Clinical Outcomes for Patients With Coronal Pelvic Obliquity After Total Hip Arthroplasty in Direct Anterior Approach

**DOI:** 10.1111/os.70060

**Published:** 2025-05-01

**Authors:** Tianyu Lai, Kaiwei Shen, Yiping Lan, Jinhua Chen, Eryou Feng

**Affiliations:** ^1^ Department of Hand and Foot Microsurgery Fuzhou Second General Hospital Fuzhou China; ^2^ Faculty of Medicine Institute of Biochemistry and Molecular Biology, Albert‐Ludwigs‐Universität Freiburg Freiburg Germany; ^3^ Xiamen ITG TaHo Rehabilitation Hospital Xiamen China; ^4^ Follow‐Up Center Fujian Medical University Union Hospital Fuzhou China; ^5^ Department of Orthopaedics, Third Ward Fujian Medical University Union Hospitail Fuzhou China

**Keywords:** acetabular component, coronal, direct anterior approach, Harris score, pelvic obliquity, total hip arthroplasty

## Abstract

**Objective:**

Abnormal pelvic coronal plane obliquity is a potential risk factor for cup instability during total hip arthroplasty. This study investigates the clinical function, acetabular cup position, leg length discrepancy, and improvement of obliquity in patients with infrapelvic obliquity after treatment with total hip arthroplasty in the direct anterior approach (DAA‐THA).

**Methods:**

A total of 987 patients who underwent DAA‐THA in the supine position from January 2017 to January 2021 were retrospectively analyzed, and 158 of them were included. The infrapelvic obliquity was classified into two types according to the direction of obliquity. Type I is when the pelvis tilts to the side of the affected lower limb, while type II is pelvic obliquity on the side of the healthy lower limb. Cases were further classified into two subtypes according to the angle of pelvic obliquity obtained: 0°–3° for type A; ≥ 3° for type B. Clinical observation and follow‐up were carried out at 1 day, 1 month, 3 months, 6 months, 1 year, and the last clinic visit (average 29 months). Standing hip radiographs were taken to measure the cup position, leg length discrepancy (LLD) and pelvic obliquity. The Harris score was used to evaluate hip function before and after surgery. Repeated measure ANOVAs were applied to compare multiple time points within groups, while the Fisher's LSD test was used for pairwise comparisons between the means of multiple samples across groups.

**Results:**

As the degree of pelvic obliquity increased for each subtype, the pre‐operative Harris score decreased and pre‐operative LLD increased. The parameters of cup position remained stable over time for each subtype. After DAA‐THA, the Harris score improved significantly and the degree of pelvic obliquity and LLD improved for each subtype (*p* < 0.001). Although the last follow‐up showed the lowest Harris score and the poorest recovery of pelvic tilt and LLD, type IB patients demonstrated the greater improvement compared to the other types.

**Conclusions:**

DAA‐THA in supine position not only significantly improves the hip function of patients with infrapelvic obliquity, but also corrects pelvic obliquity and leg length discrepancy, while maintaining stable acetabular components. For patients with infrapelvic obliquity, in which the pelvis is oblique on the affected side and the angle is more than 3°, the degree of functional improvement and correction is the greatest.

## Graphical abstract

This study identifies the positive effects of DAA‐THA on clinical function and prosthesis positioning in patients with infrapelvic obliquity.

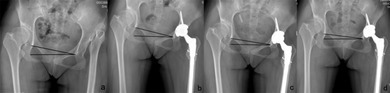

**Abbreviations**
DAA: direct anterior approach
DDH: developmental dysplasia of the hip
FAI: femoral acetabular impingement
FO: femoral offset
LLD: leg length discrepancy
OA: osteoarthritis
ONFH: osteonecrosis of femoral head
PHOA: primary hip osteoarthritis
RA: rheumatoid arthritis
THA: total hip arthroplasty

## 1 Introduction

In total hip arthroplasty (THA), failure of acetabular component positioning may lead to dislocation, impingement, pelvic osteolysis, acetabular displacement, and component wear [1]. Therefore, the primary approach to improve the longevity of components in THA is to prevent misalignment of the acetabular component, the orientation of which is dependent on the position of the bony acetabulum [2]. Pelvic obliquity will alter the normal hip joint weight‐bearing relationship and aggravate the bony deformity of the hip joint, the surrounding soft tissue contracture, and pathological progression, leading to changes in the position of the bony acetabular space. Recently, pelvic tilt occurring in the sagittal plane has received extensive attention from orthopedic surgeons as an important component of the bodily parameters and has been commonly used in the evaluation of acetabular component parameters and surgical safety in total hip arthroplasty [3]. However, not just in the sagittal plane, the disruption of pelvic equilibrium can occur in three dimensions [4], and the tilt of the coronal plane of the pelvis also has an impact on hip joint function, which is often neglected. Depending on the anatomical location, pelvic obliquity is classified into suprapelvic [5], intrapelvic [5] and infrapelvic types [6]. Pelvic obliquity is characterized by soft tissue spasms and skeletal deformities around the pelvis [5, 6]. Suprapelvic obliquity is often secondary to spinal pathologies such as scoliosis and ankylosing spondylitis. In contrast, intrapelvic obliquity is mostly secondary to morphologic changes in the hemipelvis, and infrapelvic obliquity is mostly caused by contracture deformities such as hip abduction and adduction, which is the most common type of coronal pelvic obliquity. Any disease that causes fixed abduction and adduction deformity of the hip joint will almost always eventually promote the development of infrapelvic obliquity. It is reported [7] that a pelvic obliquity greater than 6° was associated with increased cup inclination, which could lead to cup instability. In addition, a prospective study [8] demonstrates that severe pelvic obliquity affects femoral offset in patients with THA. Abnormal pelvic coronal plane obliquity can be a potential risk factor for total hip arthroplasty complications. However, fewer studies have focused on the effect of pelvic coronal obliquity on hip function.

Direct anterior approach‐total hip arthroplasty (DAA‐THA) is a superficial surgical approach that operates between the nerve interface and in the muscle space [9, 10]. This method is becoming increasingly popular with Asian surgeons [11] because of the well exposure of the acetabulum, helping surgeons with direct visualization and manual palpation to verify anatomic cup positioning. Younger age [6], medial offset discrepancy [12], large preoperative leg length discrepancy [13], and rigidity of the lumbar spine [14] were reportedly identified as important contributing factors for residual pelvic obliquity after THA. In patients with poor lumbar spine mobility [15] or a leg discrepancy greater than 1cm [16], which are the risk factors for residual postoperative pelvic obliquity, DAA‐THA demonstrates a low (< 1%) risk of instability and superior radiographic assessment compared to the traditional approach of THA. The potential reason is to easily obtain the superiority of the optimum component position, preservation of more soft tissue, and stability of the hip through the supine positioning inherent to DAA‐THA [17]. Clinical research [18] shows that pelvic positioning in the supine position leads to the more consistent orientation of the acetabular component after THA, while significant differences in pelvic tilt and rotation are seen with the patient in the lateral decubitus position. However, there are currently no studies to observe the effect of DAA‐THA in the supine position on patients with coronal pelvic obliquity. The aim of the present study is: (i) to determine the improvement in pelvic obliquity and clinical function in patients with different types of pelvic obliquity after DAA‐THA; (ii) to identify whether the cup position and leg length discrepancy corrected by DAA‐THA are affected by pelvic obliquity.

## 2 Patients and Methods

### 2.1 Ethics Approval and Consent to Participate

This study obtained approval from the Ethics Committee of Fuzhou Second General Hospital and was performed by the ethical standards of the Declaration of Helsinki of 1964. Informed consent was obtained in written form from all eligible patients.

### 2.2 Source of Cases

A total of 987 patients who underwent DAA‐THA from 2017 to 2021 in the Department of Joint Surgery were reviewed, of which 158 patients who met the criteria were included. Of these patients, 75 were type I patients (27 type IA and 48 type IB), and 83 were type II patients (33 type IIA and 50 type IIB). Seventy‐four patients were male, and 84 were female. The follow‐up period ranged from a minimum of 12 months to a maximum of 63 months, with an average of 29 months. The distribution of pre‐operative cases included 68 cases of osteonecrosis of the femoral head (ONFH), 13 cases of rheumatoid arthritis (RA), 55 cases of developmental dysplasia of the femoral head (DDH), 10 cases of primary hip osteoarthritis (PHOA), 9 cases of femoral acetabular impingement syndrome (FAI), 2 cases of hip fusion after septic hip osteoarthritis, and 1 case of hip stiffness due to hip tuberculosis. All diagnoses were given by two associate chief physicians.

### 2.3 Inclusion and Exclusion Criteria

Inclusion criteria: (1) Patients who underwent THA in the supine position at our institution, with all access methods using DAA; (2) patients with infrapelvic obliquity features as judged by imaging; and (3) those with complete basic data and imaging data.

Exclusion criteria are as follows: (1) To minimize the impact of altered spinal‐pelvic sagittal balance on this study, patients with ankylosing spondylitis and concurrent hip osteoarthritis, scoliosis, lumbar spondylolisthesis, post‐lumbar fusion, and incomplete pelvic morphology were excluded. (2) Patients with Parkinson's and other diseases with severe loss of neuromuscular stability were excluded. (3) Femoral neck fractures and the resulting osteonecrosis of the femoral head, whose condition does not reflect the true characteristics of infrapelvic obliquity, were excluded. (4) To clarify the interaction between THA and infrapelvic obliquity due to unilateral hip disease, cases with severe lesions in both hips that the operator considered to require simultaneous bilateral THA were excluded (e.g., patients with ONFH in ARCO [19] stages III and IV, patients with DDH in Crowe stage III/IV with secondary osteoarthritis causing severe pain and limitation of movement, and patients with RA with severe joint deformity and functional limitation), patients who had received THA on the contralateral side before surgery were excluded. (5) Postoperative follow‐up was less than 12 months.

### 2.4 Main Pre‐Operative Preparation

(1) Pre‐operative detailed medical history, careful physical examination, and exclusion of contraindications to surgery. (2) Check the previous spinal diseases and surgical history, assess the stability of the hip joint, and exclude the effects of spine‐pelvis sagittal imbalance to the greatest extent possible. (3) Complete the standing hip x‐ray film, observe the direction of the patient's pelvic obliquity, and measure the angle of pelvic obliquity.

### 2.5 Anesthesia and Position

General anesthesia and nerve block anesthesia were used in all patients. They were lying in a supine position on a fracture table (Hana Table, Union City, CA). Each patient's pubic symphysis was located directly at the fold markings on the table.

### 2.6 Main Procedures of the DAA‐THA

The prosthetic wear interface used intraoperatively in all cases was ceramic‐to‐ceramic or ceramic‐to‐polyethylene. Cementless hemispheric porous‐coated acetabular cups and tapered cementless stems were used in the hips of all patients. The detailed information about manufacturers of the implants used is shown in Table 1. (1) Position and exposure of the incision: the anterior superior iliac crest was used as a reference, and a mark was made 2 cm laterally and 2 cm distally, from which a surgical incision of approximately 8 cm was made along the anterior edge of the vastus tensor fasciae latae muscle toward the fibular tuberosity. The superficial fascia, vastus tensor fasciae latae, and the joint capsule were then opened layer by layer. (2) Treatment of the acetabular side: the joint capsule was incised and osteotomized at the appropriate osteotomy level and full exposure of the acetabulum. After removal of the osteochondral bone, grinding and filing of the acetabulum was carried out, and the acetabular cup was implanted at a suitable angle after satisfactory trial molding. (3) Lateral femoral management: subsequently, the soft tissue on the side of the femur was loosened, and after the loosening was in place, the patient's surgical bed was tilted back 20° above the pubic symphysis and lowered 30° below it, so that the hip joint on the operative side was kept in a posteriorly extended position and was extremely internally and externally rotated. Once the femur has been adequately exposed, the medulla can be opened, followed by sequential expansion with a medullary file. After appropriate fitting, the trial mold can be installed and the hip joint can be reset. (4) The x‐ray check: It was confirmed that the prosthesis and screw were in place at the upper margin of the obturator foramen by intraoperative C‐arm x‐ray.

**TABLE 1:** The manufacturers of the implant used in patients of various subtypes.

Manufacturers of implant	IA	IB	IIA	IIB
Zimmer Biomet (USA)	12	5	8	18
DePuy Synthes (Johnson & Johnson, USA)	9	30	16	18
Waldemar Link (Germany)	3	4	5	5
Chunli (China)	1	5	1	3
Beijing Aikang (China)	2	4	2	4
Missing	—	—	1	2
Total	27	48	33	50

### 2.7 Post‐Operative Management

A multimodal analgesic regimen covering non‐steroidal and other drugs was given postoperatively. On the first post‐operative day, all patients were encouraged to walk underweight with the assistance of a walking aid and to take standing hip x‐ray films. Rehabilitation training such as abduction and internal rotation of the affected limb was started on the second post‐operative day.

### 2.8 Observed Indicators

(1) Imaging assessment data: pre‐operative standing hip x‐ray was taken, and the cases were classified according to the direction and degree of obliquity. The clinical observation and follow‐up were performed on day 1, 1 month, 3 months, 6 months, 12 months, and the last clinic visit, respectively. (2) Functional assessment data: Harris score was used to evaluate the function of the affected hip during the preoperative and postoperative clinical observation and follow‐up at 1 month, 3 months, 6 months, 12 months, and the last outpatient visit (average 29 months).

### 2.9 Radiological Measurement Methods and Classification

**FIGURE 1:**
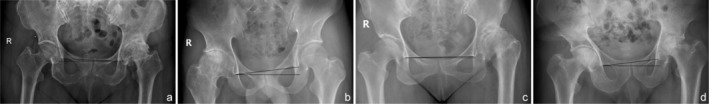
(a–d) Hip joint images in patients of various subtypes. (a) IA, the pelvis tilts toward the side of the affected limb, and the measured obliquity is less than 3°. (b) IIA, the pelvis tilts toward the side of the affected limb, and the measured obliquity is more than 3°. (c) IB, the pelvis tilts toward the side of the healthy limb, and the measured obliquity is less than 3°. (d) IIB, the pelvis tilts toward the side of the healthy limb, and the measured obliquity is more than 3°.

Both lower limbs were straightened and internally rotated by 15° to obtain a standard standing hip radiograph before surgery [20]. The obliquity was measured by the angle between the line connecting iliac crest bilaterally and the horizontal line passing through the base of the fourth lumbar vertebra on the hip orthopantomography. If the iliac spines bilaterally cannot be revealed, then the angle between the line connecting the teardrops bilaterally and the horizontal line was measured [8] Improved by the method of Lee et al. [21] The infrapelvic obliquity was divided into two types according to the direction of pelvic obliquity on imaging. Type I is pelvis inclined to the side of the affected limb, and type II is pelvis inclined to the side of the healthy limb. The cases were further divided into two subtypes according to the measured obliquity: 0°–3° for type A and ≥ 3° for type B (Figure 1), and standing hip radiographs were taken at the corresponding post‐operative time points to determine the cup abduction angle and anteversion angle and pelvic obliquity (excluding day 1).

The cup abduction angle was measured by Callaghan et al. [22] as the angle of intersection of the bilateral teardrop or sciatic spine lines with the upper and lower ends of the elliptical opening of the cup on orthopantomographic radiographs of the hip. The method from Lewinnek et al. [23] was used to determine the anteversion angle of the cup: the ratio of the short axis to the long axis of the cup was calculated on hip radiographs, and then the ratio was substituted into the inverse sine function to calculate the anteversion angle. Leg length discrepancy (LLD) was determined in centimeters by measuring the difference between the acetabular teardrops line and a bilateral line between the lesser trochanters [24]. To minimize subjective measurement errors, each radiograph was measured by two orthopedic surgeons via the Medical Image Archiving and Communication System (MICS) or Mimics 20.0, and the average of the two values was recorded. The reliability of the radiological parameters was assessed using the intraclass correlation coefficient (ICC) [25]. The interobserver kappa coefficient for classifying the type of pelvic obliquity between the two observers was 0.82 (95% confidence interval (CI) 0.71–0.95), and the intraobserver kappa coefficient was 0.92 (95% CI 0.83–0.99), both of which were considered substantial for interobserver and intraobserver variability. The reliability of both intrarater and interrater values was interpreted as excellent (≥ 0.75) for other radiological measurements evaluated.

### 2.10 Statistical Methods

SPSS 23.0 statistical software (IBM Corp, Armonk, NY, USA) was used to statistically analyze the obtained data as well as to make graphs. Measurements were expressed as mean ± standard deviation ($\bar{x}$ ± s) and repeated measures ANOVA was applied for comparison of multiple time points within groups, while the lsd‐t test was used for two‐way comparison between means of multiple samples between groups. All data were subjected to normal distribution test, and if they did not conform to normal distribution, the non‐parametric test was applied. The chi‐square test was performed for one‐way categorical variables, and if the conditions were met, the Fisher exact test was performed for single‐factor categorical variables. Then, the Fisher exact test was applied. All test levels were set at *p* = 0.05, with *p* < 0.05 indicating statistical significance.

## 3 Results

### 3.1 Comparison of Disease Composition Ratios by Subtype

The presence of varying degrees of infrapelvic obliquity was observed in all patients, and the specific number and disease characteristics were shown in the source of cases in the methods described above. Due to the small number of cases except for osteonecrosis of the femoral head (OFNH) and developmental dysplasia of the femoral head (DDH), which had a significant impact on the statistical process and resulted in the inability to use the chi‐square test and Fisher test, the other remaining diseases were grouped into the remaining diseases to facilitate the completion of the chi‐square test. The proportions of diseases included in this study showed no significant differences across the different types of pelvic tilt (*p* = 0.15, *χ* ^2^ = 24.94, df = 6), indicating that the composition ratio of diseases has not impacted the results of this study. The proportion of DDH in type IB cases (58.3%) was higher than that in other types, including type IA (29.6%), type IIA (12.1%) and type IIB patients (30%) (Table 2, the third row, *p* < 0.001). The remaining disease types in type IA and type IB cases were significantly less than those in type IIA cases (34.3%) and type IIB (42.9%) patients (Table 1, the fourth row, *p* < 0.001). The differences in OFNH composition ratios among subtypes were not statistically significant.

**TABLE 2:** Comparison of disease composition ratios by subtype.

Disease type	IA	IB	IIA	IIB	*p*
ONFH	15 (55.6%)	16 (33.3%)	17 (51.5%)	20 (40%)	> 0.5
DDH	8 (29.6%)	28 (58.3%)	4 (12.1%)	15 (30%)	< 0.001*
Other remaining diseases	4 (11.4%)	4 (11.4%)	12 (34.3%)	15 (42.9%)	< 0.001*
*p* = 0.15, *χ* ^2^ = 24.94, df = 6					
Total	27	48	33	50	

Abbreviations: DDH, developmental dysplasia of the femoral head; ONFH, osteonecrosis of the femoral head.

* Significant difference.

### 3.2 Radiographic Results About Abduction Angle and Anteversion Angle of Each Subtype

In the process of the statistics, it was found that the measurements of cup abduction angle and anteversion angle did not change significantly with the prolongation of the post‐operative period in each subtype of patients (Tables 3 and 4, *p* > 0.05). No patient was found to have loosened the prosthesis at any postoperative time point during follow‐up.

**TABLE 3:** Abduction angle of acetabular cup in patients of various subtypes.

	IA	IB	IIA	IIB
The first day after surgery	40.93 ± 0.38	38.15 ± 6.3	44.14 ± 2.03	44.91 ± 2.94
1 month after surgery	41.43 ± 0.57	39.35 ± 4.7	43.04 ± 3.14	44.33 ± 3.17
3 months after surgery	40.85 ± 0.24	39.25 ± 2.5	43.63 ± 4.15	43.41 ± 3.58
6 months after surgery	41.55 ± 0.43	40.25 ± 3.7	44.35 ± 1.57	45.01 ± 3.95
1 year after surgery	41.23 ± 0.97	38.55 ± 1.6	43.18 ± 2.33	44.21 ± 3.56
Last follow‐up visit	42.53 ± 0.12	40.35 ± 3.5	43.58 ± 1.93	44.93 ± 3.12
*F*	0.098	1.235	0.798	2.394
*p*	> 0.05	> 0.05	> 0.05	> 0.05

**TABLE 4:** Anteversion angle of the acetabular cup in patients of various subtypes.

	IA	IB	IIA	IIB
The first day after surgery	22.64 ± 3.28	20.65 ± 2.34	22.34 ± 3.6	23.13 ± 2.15
1 month after surgery	22.21 ± 4.39	21.25 ± 3.15	21.74 ± 2.4	22.90 ± 1.36
3 months after surgery	23.93 ± 3.18	22.15 ± 2.45	23.46 ± 1.5	23.30 ± 3.04
6 months after surgery	23.43 ± 2.94	21.13 ± 1.64	22.63 ± 2.5	22.45 ± 2.78
1 year after surgery	24.13 ± 3.48	21.15 ± 3.14	23.14 ± 2.6	24.01 ± 2.76
Last follow‐up visit	23.34 ± 4.01	22.32 ± 4.65	23.17 ± 2.5	23.58 ± 2.87
*F*	0.827	1.352	1.485	3.410
*p*	> 0.05	> 0.05	> 0.05	> 0.05

### 3.3 Harris Score in Patients of Various Subtypes

**FIGURE 2:**
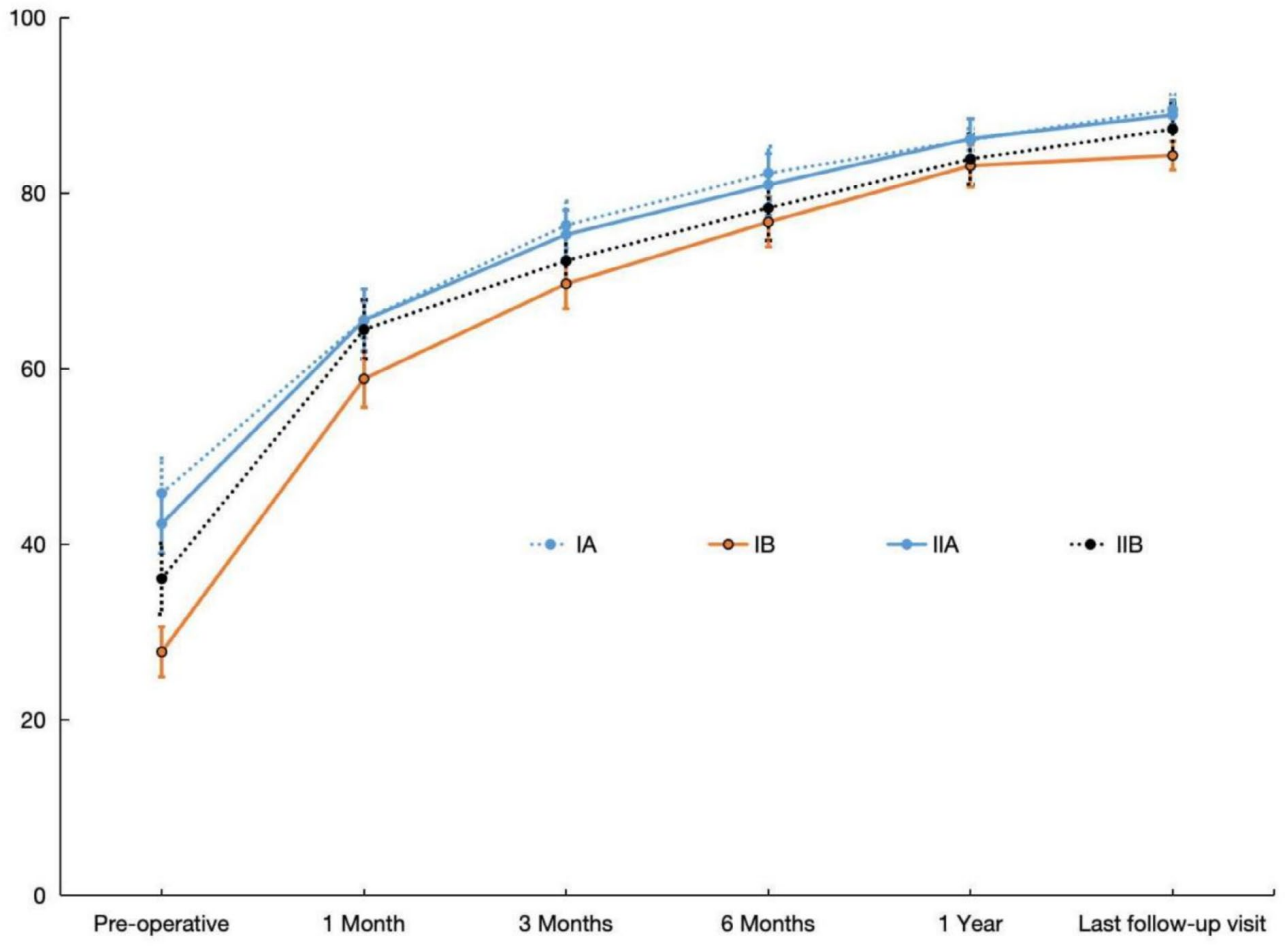
Changes in the degree of Harris scores in each subtype of patients during the treatment.

Statistical results showed that the pre‐operative Harris score decreased as the degree of pelvic obliquity increased. With the post‐operative time progressed, the Harris score improved significantly in all subtypes compared with the pre‐operative time, and the difference between time points was statistically significant (*p* < 0.001), showing a time effect (Table 5). At each time point, the difference in scores between groups was statistically significant (*p* < 0.05), showing an interaction effect (Table 5). Asterisk in Table 6 indicates that at the 1‐year postoperative follow‐up, the mean functional scores of type IB patients were less than those of type IA and IIA patients, and the difference was statistically significant (*p* < 0.05). Although the last follow‐up Harris score was the lowest, type IB patients showed greater improvement compared to the other types (Figure 2).

**TABLE 5:** Results of ANOVA for repeated measurements of Harris scores in patients of each subtype.

SOV	SS	DOF	Mean square	*F*	*p*
Between‐group (group)	7826.93	3	2608.98	268.38	0.000
Within‐group (Time)	249307.12	5	49861.42	5982.89	0.000
Group × Time	3272.48	15	218.17	25.78	0.000

Abbreviations: DOF, degree of freedom; SOV, source of variation; SS, sum of squares of deviation from mean.

**TABLE 6:** Comparison of Harris scores of patients by subtype.

	IA	IB	IIA	IIB	*F*	*p*
Pre‐operative	45.78 ± 3.96	27.71 ± 3.00	42.36 ± 3.47	36.06 ± 4.05	185.08	< 0.05
1 month after surgery	65.52 ± 2.06	58.81 ± 3.21	65.48 ± 3.60	64.42 ± 3.36	43.25	< 0.05
3 months after surgery	76.30 ± 2.71	69.63 ± 2.91	75.24 ± 2.81	72.26 ± 2.8	42.77	< 0.05
6 months after surgery	82.22 ± 3.04	76.69 ± 3.67	81.00 ± 3.55	78.28 ± 3.71	18	< 0.05
1 year after surgery	86.04 ± 1.22	81.25 ± 1.36*	86.06 ± 1.22	84.48 ± 1.34	122.36	< 0.05
Last follow‐up visit	89.48 ± 1.70	83.5 ± 1.53	88.64 ± 1.41	86.64 ± 1.42	121.34	< 0.05
*F*	1188.84	2666	1275.15	1963.7		
*p*	< 0.001	< 0.001	< 0.001	< 0.001		

* Significant difference.

### 3.4 Degree of Pelvic Obliquity in Patients of Each Subtype

**FIGURE 3:**
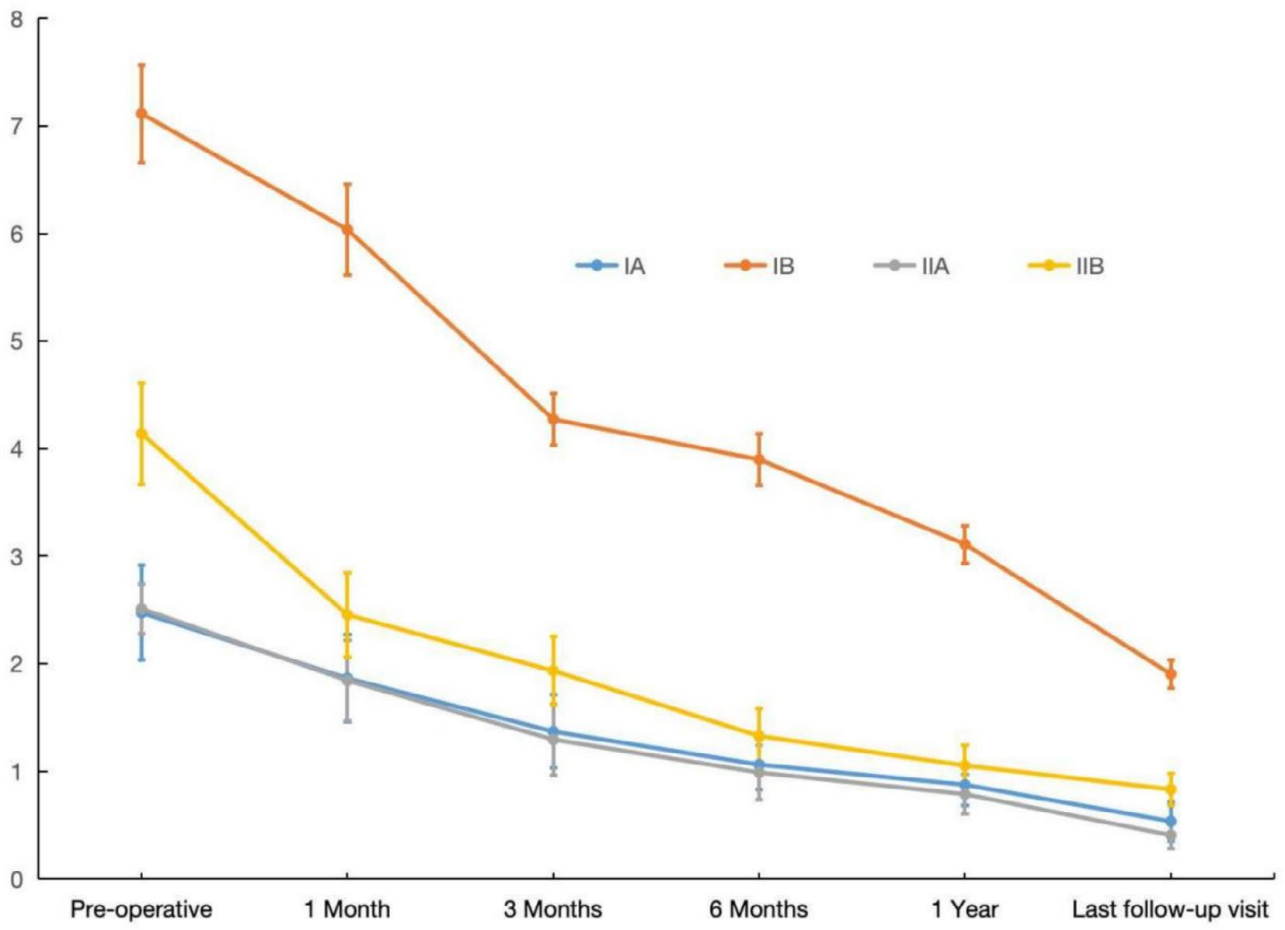
Changes in the degree of pelvic obliquity angle in each subtype of patients during the treatment.

The statistical results suggest that with the prolongation of post‐operative time, the pelvic obliquity of patients in each subtype improved to different degrees compared with that before surgery, and the difference between time points within the group was statistically significant (*p* < 0.001), showing a time effect (Tables 7 and 8). At each time point, the mean pelvic obliquity of patients of type IB was greater than that of other subtypes, and the difference was statistically significant (*p* < 0.05), also showing an interaction effect (Tables 7 and 8). However, it is easy to find that type IB patients showed the greater improvement of post‐operative pelvic obliquity compared to the other types (Figure 3).

**TABLE 7:** Results of ANOVA for repeated measurements of pelvic obliquity angle in patients of each subtype.

SOV	SS	DOF	Mean square	*F*	*p*
Between‐group (group)	1604.60	3	534.87	3878.69	0.000
Within‐group (time)	960.46	5	192.09	2364.06	0.000
Group × Time	207.36	15	13.82	170.13	0.000

Abbreviations: DOF, degree of freedom; SOV, source of variation; SS, sum of squares of deviation from mean.

**TABLE 8:** Comparison of the degree of pelvic obliquity in patients of each subtype.

	IA	IB	IIA	IIB	*F*	*p*
Pre‐operative	2.47 ± 0.44	7.11 ± 0.45	2.51 ± 0.42	4.14 ± 0.47	950.46	< 0.05
1 month after surgery	1.86 ± 0.40	6.02 ± 0.43	1.84 ± 0.38	2.45 ± 0.39	1064	< 0.05
3 months after surgery	1.36 ± 0.33	4.27 ± 0.24	1.30 ± 0.33	1.93 ± 0.32	849.81	< 0.05
6 months after surgery	1.36 ± 0.33	3.90 ± 0.24	0.98 ± 0.25	1.33 ± 0.25	1401.48	< 0.05
1 year after surgery	0.87 ± 0.19	3.11 ± 0.17	0.80 ± 0.18	1.05 ± 0.18	1621.04	< 0.05
Last follow‐up visit	0.59 ± 0.18	1.9 ± 0.13	0.41 ± 0.12	0.83 ± 0.15	948.74	< 0.05
*F*	168.22	2025.95	246.67	826.95		
*p*	< 0.001	< 0.001	< 0.001	< 0.001		

### 3.5 Leg Length Discrepancy in Patients of Various Subtypes

**FIGURE 4:**
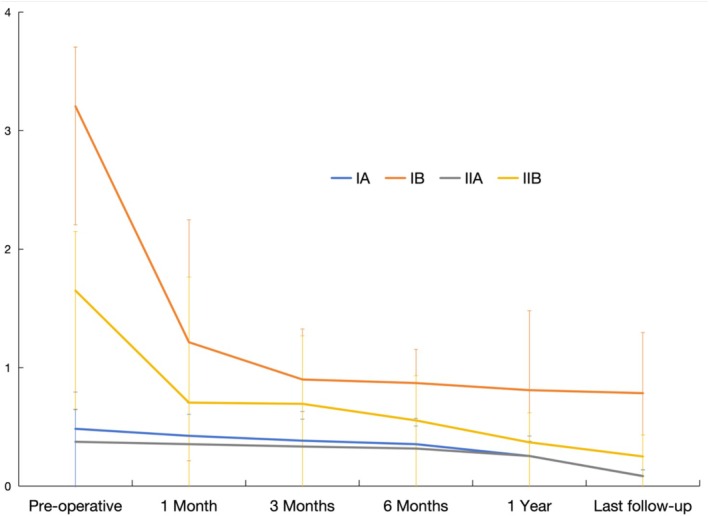
Changes in the leg length discrepancy in each subtype of patients during the treatment.

First, the greater pre‐operative pelvic obliquity angles exacerbated LLD, which was more prominent when the pelvis inclined to the side of the affected limb. Pre‐operative LLDs in all subtypes could be corrected after DAA‐THA, and the difference between time points within the group was statistically significant (*p* < 0.001), showing a time effect (Tables 9 and 10). Among these, type IB patients showed the greater improvement in LLD compared to other types. At each time point, the mean LLD of type IB patients was greater than that of other subtypes, and the difference was statistically significant (*p* < 0.05), also showing an interaction effect (Tables 9 and 10, Figure 4).

**TABLE 9:** Results of ANOVA for repeated measurements of leg length discrepancy in patients of each subtype.

SOV	SS	DOF	Mean square	*F*	*p*
Between‐group (group)	157.59	1	380.66	805.07	0.000
Within‐group (time)	118.43	2.52	47.00	51.49	0.000
Group × Time	105.81	7.57	14.00	15.33	0.000

Abbreviations: DOF, degree of freedom; SOV, source of variation; SS, sum of squares of deviation from mean.

**TABLE 10:** Comparison of leg length discrepancy in patients of each subtype.

	IA	IB	IIA	IIB	*F*	*p*
Pre‐operative	0.48 ± 0.31	3.20 ± 0.51	0.37 ± 0.27	1.65 ± 0.54	45.38	< 0.05
1 month after surgery	0.42 ± 0.28	1.21 ± 1.03	0.35 ± 0.25	0.71 ± 1.06	8.51	< 0.05
3 months after surgery	0.38 ± 0.25	0.90 ± 0.43	0.33 ± 0.23	0.69 ± 0.57	14.79	< 0.05
6 months after surgery	0.35 ± 0.22	0.87 ± 0.28	0.32 ± 0.19	0.55 ± 0.38	29.36	< 0.05
1 year after surgery	0.25 ± 0.17	0.81 ± 0.67	0.25 ± 0.12	0.37 ± 0.25	17.41	< 0.05
Last follow‐up visit	0.09 ± 0.05	0.78 ± 0.51	0.08 ± 0.05	0.25 ± 0.18	50.16	< 0.05
*F*	1338.45	1235.25	1325.45	976.10		
*p*	< 0.001	< 0.001	< 0.001	< 0.001		

### 3.6 Imaging Data of a Typical Type IB Case Are Shown Below (Figure 5a–d)

**FIGURE 5:**
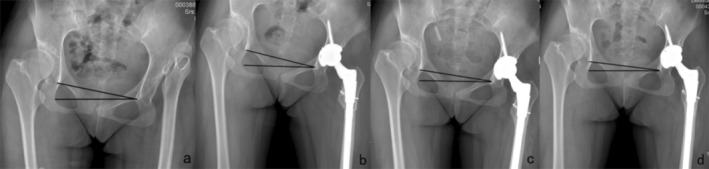
(a–d) The patient, female, 22 years old, with a pre‐operative diagnosis of DDH Crowe type IV and combined type IB infrapelvic obliquity. DAA‐THA was performed successfully. (a) The pre‐operative radiograph shows significant pelvic obliquity to the affected side. (b) The pelvic obliquity is mildly improved after 1 month of DAA‐THA compared to the pre‐operative radiograph. (c) Six months postoperative radiographs showed a significant improvement in pelvic obliquity of the patient. (d) At the last follow‐up, the degree of pelvic obliquity on radiographs continued to be improved, but some pelvic obliquity remained.

## 4 Discussion

According to incomplete statistics in 2011, there were about 8.12 million ONFH and 16.05 million DDH patients in China, with a total prevalence rate of 2.245% [26]. By 2050, nearly 10 million people in Asia are expected to suffer from various types of hip disorders [27, 28]. Therefore, severe coronal pelvic obliquity becomes more common, and it is an unavoidable challenge for surgeons to face during THA. The direct anterior approach can be a great strategy for the treatment of coronal pelvic obliquity in THA. Intraoperative pelvic motion during acetabular cup implantation is a major factor affecting acetabular cup positioning [29]. THA in the supine position is associated with less disruption of pelvic equilibrium and a better acetabular cup placement than THA performed in the lateral decubitus position [30]. Lesser soft‐tissue damage [30] in DAA‐THA helps to correct pelvic obliquity with soft tissue spasms. Although few studies focus on the effect of DAA‐THA on coronal pelvic obliquity, this study fills a gap in this area. We found that as the degree of pelvic obliquity increased for each subtype, both clinical function and LLD became worse. After DAA‐THA, the parameters of cup position remained stable over time for each subtype. The Harris score improved significantly, and both the degree of pelvic obliquity and LLD showed improvement in each subtype. Although the last follow‐up revealed the lowest Harris score and the poorest recovery of pelvic tilt and LLD, type IB patients demonstrated greater improvement compared to the other types.

### 4.1 Analysis of the Bias Caused by DDH‐Derived OA in Patients With Pelvic Obliquity Included in This Study

The impact of hip contracture and subluxation on the direction of pelvic obliquity in DDH patients has been reported [31]. Hips affected by OA secondary to developmental dysplasia of the hip show greater pelvic obliquity compared to those with osteonecrosis of the femoral head [32]. In this study, DDH patients indeed make up a higher proportion in Type IB within the total inclusion of the DDH population, but this does not potentially affect the reliability of our results. Firstly, despite the higher number of Type IB DDH patients, the disease composition ratio shows no statistically significant differences between four pelvic tilt types, which minimizes potential research bias caused by DDH. Secondly, the pathological progression of pelvic tilt due to hip disease eventually leads to either shortening of the affected limbs (tilting toward the affected side) or a pain‐avoidance mechanism (tilting toward the unaffected side) [31]. Therefore, it is necessary to ensure that different types of hip diseases are included with an even distribution, as this does not impact bias. Thus, it is feasible to include DDH‐derived OA and others as equivalent diseases.

### 4.2 Analysis of Acetabular Cup Parameters in Patients With Pelvic Obliquity After DAA‐THA

The position of the bony acetabulum determines the placement of the acetabular prosthesis. After accurate 3D reconstruction and digital transformation of 20 selected normal pelvic imaging data in a horizontal position without bony deformity, Min Lingtian et al. [33] found that pelvic obliquity would have a sharp effect on the abduction angle of the acetabulum, but not on its anteversion angle. It has been found that patients with infrapelvic obliquity on the shortened side of the limb and a tilt angle of > 6° (classified as type IC in this study) have a less bony acetabular abduction angle and a larger cup abduction angle than the other subtypes, and it is therefore proposed that the cup placement angle should be reduced compared with the target abduction angle during posterior‐lateral total hip arthroplasty [8]. The reason is that pelvic obliquity is partially corrected in this subtype of pelvic obliquity after total hip arthroplasty, and this can lead to excessive abduction and perpendicularity of the cup, which is not favorable to the stability of the cup.

However, in this study, after the pelvic obliquity was improved, the cup abduction angle and anteversion angle did not change accordingly. The possible reasons for the opposite results in this study to the above studies are as follows. First of all, the pelvic position during DAA‐THA is stable in the supine position, less prone to additional rotation and tilt, and easier for the operator to detect and correct even if the position changes. More importantly, in the supine position, the operator can place the acetabular cup more visually and easily by using a fixed marker such as the sagittal line of the trunk or the edge of the surgical bed as a reference. In this case, the acetabular cup is placed in a position that better fits the acetabulum in the physiological state of the pelvis. When the pelvic obliquity is corrected, there is no need to increase or decrease the angle of the cup, whereas when total hip arthroplasty is performed in the posterior lateral position, the pelvis may be distorted due to the unclear positioning reference and the influence of gravity, which makes it more difficult to determine the position of the cup. Secondly, there are many clinical methods used to determine the position of the acetabular component, such as transverse ligament positioning and acetabular rim anatomic marker positioning, but infrapelvic obliquity is often accompanied by anatomic variation and soft tissue abnormalities, which makes the positioning of the cup more difficult in cases of severe hip disease combined with pelvic obliquity. The studies by Min Lingtian [33] did not specify the specific type of pelvic obliquity and were performed under in vitro conditions, so it is difficult to correctly and truly reflect the influence of infrapelvic obliquity on the positioning of the cup under certain conditions, and maybe the conclusions reached are somewhat limited. The operators in this study were senior practitioners who had passed the DAA‐THA learning curve and had extensive experience in arthroplasty, which reduces the risk of pelvic obliquity. In summary, this may be the reason why the cup position parameters remained stable in the results of this study. However, the effects of the actual mechanisms of infrapelvic obliquity on acetabular component orientation remain unknown. Therefore, orthopedic surgeons need more comprehensive studies to properly understand as well as address the relationship between acetabular orientation and infrapelvic obliquity in order to more accurately and safely implant acetabular cups.

### 4.3 Analysis of the Obliquity Degree in Patients With Pelvic Coronal Obliquity After DAA‐THA

**FIGURE 6:**
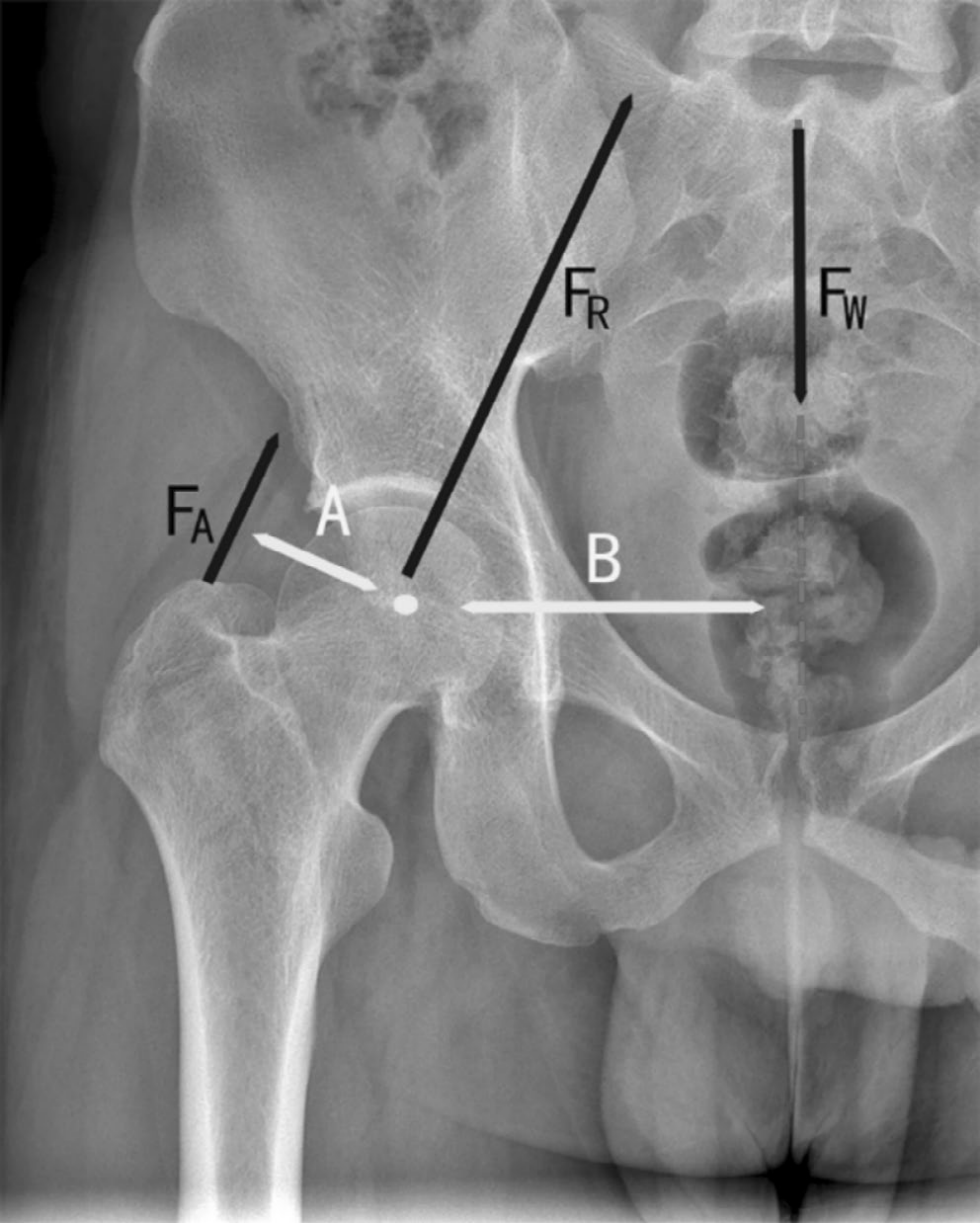
Schematic diagram showing the static biomechanical relationship of the hip joint in single‐leg stance. The weight vector (Fw) is derived from the center of gravity, oriented perpendicular to the ground, and is balanced in a single‐leg stance by the abductor force, which is related to the abductor arm (FA). At this point, the weight of the weight‐bearing leg plus the size of the body weight is approximately equal to the body weight. The weight lever arm (B) is defined as the vertical distance between the center of hip rotation and the weight vector. The vertical distance between the center of hip rotation and the abductor force vector is then the abductor lever arm (A), which can be equated to the femoral eccentric distance (FO).

The results of this study suggest that the pelvis is less likely to return to normal in patients with more than three degrees of pre‐operative pelvic obliquity on the affected lower limb. The hip joint is one of the largest and most stable joints in the human body, and the forces in all directions are in a dynamic balance. Friedrich Pauwels [34] introduced his newer biomechanical model in 1976, which included the conversion of a portion of tensile stress into compressive stress by the iliotibial bundle acting as a tension band and further supporting the function of the abductor muscles. The static biomechanical model describes hip loading in the single‐legged stance phase of normal gait (Figure 6). Femoral offset (FO) is defined as the vertical distance from the center of rotation of the femoral head to the long axis of the femoral stem [35]. The larger the FO, the longer the abductor arm, and the stronger the abductor muscle strength. Conversely, a shorter FO means that higher adductor muscle strength is required to balance the weight. A sustained increase in abductor strength requirements can lead to fatigue and even spasm of the abductors, and the resulting increased hip reaction forces can also have a very negative effect on the acetabular component. It has been shown in the literature [11] that the FO of IC type (pelvis inclined to the affected side and the angle of deviation > 6°) was less than that of the rest of the subtypes (*p* < 0.05), which could easily lead to abnormal proximal femoral or acetabular morphology after THA [36]. In the IC type with smaller FO values in this study, this change may lead to increased tension and contracture of the abductor muscle. If the FO is not accurately reconstructed intraoperatively, the increased need for abductor strength will result in the affected hip being further abducted and thus becoming lower. This may exacerbate the development of IC (i.e., greater type I) infrapelvic obliquity. However, it remains unclear why some of the type II infrapelvic obliquity remains. Therefore, based on the above speculation, the type IB infrapelvic obliquity in this study may be considered to be irreversible and fixed to a certain extent and difficult to be completely corrected by total hip arthroplasty, thus explaining the difficulty in recovering the degree of pelvic obliquity in the type IB patients in this study compared with the other subtypes.

### 4.4 Analysis of Clinical Function in Patients With Infrapelvic Obliquity After DAA‐THA

The results of this study suggest that patients with more than three degrees of pre‐operative pelvic obliquity on the affected lower limb have greater difficulty in recovering function after surgery. In addition, the combination of post‐operative changes in pelvic obliquity in each type of patient suggests that there is a negative correlation between pelvic obliquity and clinical function.

In the present study, we notice that the number of DDH in IB cases is higher than that in other subtypes. It has been shown [37] that DDH patients have significantly reduced femoral coverage, increased neck‐stem angle, and lateralized hip rotation center. In addition, patients with DDH tend to have an excessively anterior femoral neck, causing the greater trochanter to move backwards and reducing the abductor arm [38]. Therefore, the less physiological FO value in patients with DDH may exacerbate the development of type IB infrapelvic obliquity. Furthermore, it is difficult to correct the pelvic obliquity deformity in type IB patients, and the center of gravity of the body falls on the affected limb for a long period of time, which may also exacerbate the degree of difficulty in restoring function in the postoperative period.

Compared with the traditional approach to total hip arthroplasty, DAA‐THA has the advantages of less soft tissue damage, less bleeding, etc. [39, 40]. This view has been supported by objective indicators such as biological and serological tests [41, 42]. At the same time, several studies have shown that less analgesic medication and a shorter mean time out of bed [43] were used for patients after DAA‐THA [44]. Therefore, rapid post‐operative recovery is an advantage of DAA‐THA [45]. The results showed that although the overall functional recovery was poor in patients with type IB, the absolute Harris scores of patients with each subtype improved with time after surgery compared with the preoperative period (*p* < 0.001), and the rate of functional improvement was particularly prominent at the 1‐month follow‐up, which further highlighted the advantages of DAA‐THA in the treatment of pelvic obliquity in terms of rapid recovery. However, there is still a need for longer follow‐up after DAA‐THA in the bulk of patients with pelvic obliquity, so that a more comprehensive picture of the association between pelvic obliquity and patients' functional recovery can be obtained, which may also provide guidance to clinicians in directing the postoperative rehabilitation of this type of patient.

### 4.5 Strength and Limitation

Our study has certain advantages. First, the impact of pelvic coronal plane obliquity on the stability of prosthetic implants in THA has received relatively less attention compared to pelvic sagittal plane obliquity, making this topic novel. Second, this study classified patients based on the direction and angle of pelvic obliquity, and the outcomes observed included both clinical function and radiological indicators, making the research objectives more clinically relevant. For statistical analysis, we used repeated measures ANOVA. This method is ideal for longitudinal data and, compared to independent sample ANOVA, can reduce the impact of individual differences and increase statistical power.

In terms of limitations, although the inclusion and exclusion criteria for this study minimized the effect of changes in the sagittal balance of the spine and pelvis due to several diseases, the exclusion criteria are likely incomplete due to the limitations of current knowledge of the diseases. It is unknown whether there are additional musculoskeletal disorders that alter the pelvis in the sagittal and vertical axes, and these changes may have unexplained effects on this study. In addition, the short follow‐up period of this study does not allow complete proof that the pelvic status of the patient at the last follow‐up remains stable. This requires dynamic observation and analysis over a longer time axis, but it has also been shown that the degree of change in coronal pelvic obliquity is not significant for a significant period after THA [46]. Also, this is a retrospective study, and the monocentric nature of the data presents the possibility of selection bias. Since the patient's willingness to undergo CT examination at the postoperative follow‐up was not strong, we used plain films to measure the acetabular prosthesis position parameters. However, the value of radiological measurements in assessing acetabular prosthesis orientation and the degree of pelvic obliquity has not been uniform to date. All of these factors may have influenced the results of the current study.

## 5 Conclusion

DAA‐THA in supine position not only significantly improves the hip function of patients with infrapelvic obliquity, but also corrects pelvic obliquity and leg length discrepancy, while maintaining stable acetabular components. For patients with infrapelvic obliquity, in which the pelvis is oblique on the affected side and the angle is more than 3°, the degree of functional improvement and correction is the greatest. For these patients, surgery should be performed as early as possible after excluding contraindications to surgery. Appropriate release of abductor spasm, as well as accurate reconstruction of the femoral offset are important for them to maintain hip stability, pelvic balance, and improve hip function.

## Author Contributions

Eryou Feng and Tianyu Lai designed and conceived the paper. Kaiwei Shen was responsible for follow‐up, data collection, analysis, and processing. Yiping Lan modified the image. Jinhua Chen provided guidance on the construction of statistical methods for data. Kaiwei Shen provided some ideas and creativity for the paper and contributed to the data processing. Kaiwei Shen and Tianyu Lai were substantially modified. All authors read and approved the final manuscript.

## Ethics Statement

This study obtained the approval from the Ethics Committee of Fuzhou Second General Hospital (approval number: 2023072) and was performed in accordance with the ethical standards of the Declaration of Helsinki of 1964. Informed consent was obtained in written form from all eligible patients.

## Consent

The authors have nothing to report.

## Conflicts of Interest

The authors declare no conflicts of interest.

## Data Availability

The datasets used and/or analyzed during the current study are available from the corresponding author on reasonable request; please contact the co‐first author, Tianyu Lai. Administrative permission was received from Fuzhou Second General Hospital (Cangshan District, Fuzhou, China) to access the medical records.
